# As Effective as You Perceive It: The Relationship Between ChatGPT’s Perceived Effectiveness and Mental Health Stigma

**DOI:** 10.3390/bs15121724

**Published:** 2025-12-12

**Authors:** Scott N. Hannah, Deirdre Drake, Christopher D. Huntley, Joanne M. Dickson

**Affiliations:** 1 School of Arts and Humanities, Psychology Discipline, Edith Cowan University, Joondalup, WA 6027, Australia; shannah0@our.ecu.edu.au (S.N.H.);; 2 School of Medicine, University of Liverpool, Liverpool L69 7ZX, UK; 3 Centre for Precision Health, Edith Cowan University, Joondalup, WA 6027, Australia

**Keywords:** artificial intelligence (AI), AI chatbot, ChatGPT, anticipated stigma, self-stigma, mental health

## Abstract

Individuals are increasingly using artificial intelligence chatbots, such as ChatGPT, to seek conversational support for their personal mental health difficulties. Heightened concerns about mental health stigma may make anonymous, on-demand chatbot interactions more appealing for some than traditional face-to-face support. This study examined if using ChatGPT-4 for personal mental health difficulties is associated with two distinct forms of stigma, anticipated stigma and self-stigma. Our main aim was to investigate if the perceived effectiveness of ChatGPT use for mental health issues mediates the relationship between ChatGPT usage and anticipated stigma and self-stigma. The sample comprised 73 participants, mostly undergraduate psychology students. Participants completed online self-report measures to assess ChatGPT usage for mental health purposes, perceived effectiveness of ChatGPT for mental health issues, and anticipated stigma and self-stigma. Perceived effectiveness of ChatGPT was significantly and positively correlated with ChatGPT usage, and significantly negatively correlated with reduced anticipated stigma. Cross-sectional analyses found that perceived effectiveness significantly mediated the relationship between ChatGPT use and anticipated stigma, but not for self-stigma. The results indicate that ChatGPT use, when perceived as effective, is associated with a reduction in anticipated stigma concerning mental health issues. More research is now needed in this emerging area to inform best practice on the use of AI aids for mental health issues.

## 1. Introduction

The stigma surrounding mental health disorders and symptoms presents a significant challenge for those who experience it (Fox et al., 2018). The disparity between the prevalence of mental illness and the proportion of individuals receiving care (Keynejad et al., 2021) is worsened by stigma (Dua et al., 2011). Difficulty recognizing poor mental health symptoms, avoiding treatment, and anticipated discrimination are significant obstacles to seeking help for mental health difficulties (Henderson et al., 2013). Stigma also exacerbates mental health symptoms, further perpetuating negative cycles that can decrease overall personal well-being (Boyd et al., 2014).

Two key forms of stigma are implicated in mental health. Anticipated stigma is defined as the perception that others would judge or discriminate against you if you were to have mental health difficulties (Docksey et al., 2022; Quinn et al., 2015). This form of stigma is understood as the initial point where individuals with and without mental health difficulties start developing negative opinions about mental health (Fox et al., 2018). Anticipated concerns over being labeled by these negative prejudiced perceptions can increase stress, leading to negative psychological and somatic symptoms (Lannin et al., 2015; Link & Phelan, 2006). Self-stigma is defined as feeling devalued after internalizing the perceived negative stereotypes associated with anticipated stigma regarding mental health difficulties (Lannin et al., 2015; Quinn & Earnshaw, 2013). Specifically, this internalization process often triggers rumination, perpetuating critical negative self-evaluation (Dickson et al., 2019; Jennings et al., 2015). Self-stigma is thought to diminish motivation, exacerbate depressive cycles and ultimately reduce help-seeking behaviors (Dickson & Moberly, 2013).

The degree of stigma one feels can vary by age and gender, accounting for 22% of the variance in stigma experiences (Fox et al., 2018). Individuals aged 40 years or over generally hold less stigmatized views than those below 40 years of age, perhaps due to increased contact with those diagnosed with mental health issues (Bradbury, 2020; Gonzalez et al., 2005). Additionally, younger adults can hold increased stigmatized views due to peer influences and cultural norms (Griffiths et al., 2014; YMCA, 2016). University students are thought to be particularly vulnerable to anticipated and self-stigma due to the competitive and ability-oriented university culture (Guarneri et al., 2019).

Research has shown that the anticipated stigma university students face is deeply interconnected with academic competence (Wada et al., 2019). Due to fears of judgment and potential loss of educational status, students often conceal mental health issues and avoid seeking help. This self-isolation further reduces their sense of belonging and limits opportunities for open discussion that could decrease stigma (Wada et al., 2019). Research suggests that ease of access and stigma concerns may make Artificial Intelligence (AI) chatbot-delivered mental health support more appealing for student users (Rackoff et al., 2025). Other barriers to university students using more traditional forms of help compared to AI chatbot-delivered mental health support have been financial constraints, limited time, anticipated and self-stigma (Rackoff et al., 2025). Therefore, with the fast and emerging rise of AI technology, our research aimed to investigate whether stigma was associated with university students’ mental health help-seeking behaviors via AI chatbots.

AI is increasingly being used for a host of reasons, including for mental health purposes (Haque & Rubya, 2023; Li et al., 2024). Individuals open to online help-seeking are now having conversations with automated AI chatbots about their mental health difficulties, as a convenient and cheap alternative solution for mental health support (Abd-Alrazaq et al., 2020; Caldarini et al., 2022). AI platforms such as OpenAI’s ChatGPT are now recognized as a potential digital mental health service option offering conversational support to its users (Boucher et al., 2021).

Chatbots for mental health support are particularly appealing to individuals experiencing higher stigma-related barriers (Miles et al., 2021), possibly because they allow individuals to avoid stigmatizing labels associated with traditional care (Kosyluk et al., 2024) and foster increased self-disclosure through anonymity and reduced social judgment (Lucas et al., 2017). Despite not specifically designed for therapy purposes, individuals are increasingly using AI chatbots such as ChatGPT for support with their mental health (Moore et al., 2025). It is therefore important to examine the relationships between general-purpose AI chatbot usage for mental health difficulties and stigma.

A systematic review of AI chatbot use in mental health contexts noted that while chatbots have significantly reduced depressive symptoms, the clinical significance was limited (Abd-Alrazaq et al., 2020). However, other findings suggest that AI tools, including ChatGPT, could assist clinical and non-clinical populations in supporting their mental health, stigma concerns and promoting treatment adherence (Alanezi, 2024; Gardiner et al., 2017; Li et al., 2024). Specifically, two mental health educators and practicing psychologists reviewed ChatGPT’s responses, finding them empathetic, educational, accurate, and relevant (Maurya et al., 2023). Contrary to this, many argue that chatbots lack essential empathy and cannot provide comprehensive mental health support (Brown & Halpern, 2021; Miner et al., 2017). The present research, for the first time, aims to investigate potential associations between real-world ChatGPT use and anticipated stigma and self-stigma in relation to mental health difficulties and whether perceived effectiveness is an important variable that, in part, may explain this relationship.

Perceived effectiveness concerns one’s perceptions or beliefs about the impact or outcome an intervention or activity will yield and fits within the Theory of Planned Behavior (TPB; Ajzen, 1991). The TPB posits that positive or negative evaluations of performing a behavior are determined by beliefs about a behavior’s outcome(s), called expectancies, and the subjectively rated value of those outcomes. As such, TPB and perceived effectiveness can help explain phenomena such as the placebo effect. If people believe or perceive that they are receiving an effective treatment, they are more likely to experience its effects, even if there is no active agent (Montgomery & Kirsch, 1997). This expectancy mechanism has been shown to be a powerful predictor of mental health outcomes. For instance, in a controlled study, participants who knew they were receiving an active antidepressant (citalopram) experienced significantly greater reductions in depressive symptoms compared to those who believed they might receive a placebo, despite all receiving the same active medication (Rutherford et al., 2017). This finding highlights the critical role of expectancy and perception in mediating mental health intervention effectiveness. If individuals believe ChatGPT is effective in supporting their mental health, they may perceive less anticipated and self-stigma because the interaction is anonymous, safe, and non-judgmental. Conversely, if they view ChatGPT as ineffective, their negative expectations of judgment may persist. To help understand the acceptance of using AI technologies, an influential model has been created, the Artificially Intelligent Device Use Acceptance (AIDUA) model (Gursoy et al., 2019), which builds on the earlier and more general Theory of Planned Behavior (TPB; Ajzen, 1991) model. In the context of the AIDUA model, perceived effectiveness aligns with the secondary appraisal stage (specifically, performance expectancy), reflecting users’ cognitive evaluations of whether AI can provide meaningful mental health support. Stigma-related concerns map onto the core affect (emotion) stage, indicating that emotional responses such as discomfort, anticipated judgment, or anxiety may shape behavioral intentions toward adopting or rejecting ChatGPT for mental health use. Research testing the Artificially Intelligent Device Use Acceptance (AIDUA) model (Gursoy et al., 2019), an extension of the Theory of Planned Behavior (Ajzen, 1991), suggests that positive emotional attitudes, including expectancy, predict AI chatbot use (Ma & Huo, 2023).

With the fast-emerging impact of AI in society, there are pressing calls for more research to better understand the implications of using AI chatbots for mental health purposes (Bewersdorff et al., 2023; Kaplan et al., 2023). Therefore, the present study sought to examine the relationships between ChatGPT usage, perceived effectiveness of using AI for mental health issues, and anticipated stigma and self-stigma.

### The Present Study

Considering ChatGPT’s increasing use for mental health support, we first aimed to investigate potential associations between ChatGPT use for mental health purposes and its perceived effectiveness, anticipated and self-stigma. Our second aim was to investigate whether perceived effectiveness mediates the relationship between ChatGPT use and stigma. We first hypothesized there would be positive significant correlations between ChatGPT use and anticipated stigma and self-stigma, as people who feel stigmatized will have an expectancy that the tool may provide non-judgmental responses and advice, with ChatGPT usage as one avenue. It would be expected that people using ChatGPT for mental health support believe in its utility. Therefore, we hypothesized that perceived effectiveness of ChatGPT would indirectly mediate the relationship between ChatGPT usage and anticipated stigma and self-stigma, respectively, consistent with the AIDUA model predictions, as expectancy effects may help shape beliefs about stigma.

## 2. Method

### 2.1. Participants

The sample consisted of 73 participants, including 62 university students (84.9%) and 11 community participants (15.1%) aged 18–62 years, with a mean age of 29.56 years (SD = 9.89 years). The sample comprised 16 males (21.9%) and 56 females (76.7%) and one participant who identified as non-binary/third gender. There was no significant difference between the university sample and the community sample on the main study variables (all *p*s > 0.05), indicating the suitability of combining the samples. The 73 participants that met the criteria of reported ChatGPT use or current ChatGPT use were drawn from a larger sample (*n* = 397), which included people who had never used ChatGPT for mental health difficulties. Participants who indicated that they do not currently use and have not previously used ChatGPT for help with their own mental health difficulties were excluded from the study. Additionally, participants who indicated that they had never used but would be open to using ChatGPT for help with their mental health difficulties if they had a mental health problem were also excluded from the study. Therefore, the percentage of participants meeting the criteria for the present study was 18.4% (ratio 73:324).

### 2.2. Materials

#### 2.2.1. Demographic Questionnaire

Participants completed demographic items including age, gender and highest educational qualification.

#### 2.2.2. ChatGPT Usage and Perceived Effectiveness

For the purpose of this research, and because of the lack of established measures in this emerging area of research, single individual items were designed to assess ChatGPT usage and its perceived effectiveness. The items were piloted with five students and validated by the research team. The first item asked ‘*Have you ever used, or do you currently use, ChatGPT for help with your own mental health difficulties? Please select either Yes or No*’ (item 1). Participants who met the criteria for the present study rated two further items about their ChatGPT use, using 5-point Likert scales to assess the extent of ChatGPT usage (item 2) and its perceived effectiveness (item 3). Participants rated, ‘*To what extent have you used ChatGPT to help with your own mental health difficulties?*’ The options participants could describe were coded from 1 to 5 (1 = ‘*once*’, 2 = ‘*two times or more*’, 3 = ‘*5 times or more*’, 4 = ‘*10 times or more*’, 5 = ‘*20 times or more*’). The *perceived effectiveness* variable was measured by asking participants, ‘*To what extent do you think that engaging with ChatGPT has helped with your own mental health difficulties?*’ Participants rated the items that best described them on a scale ranging from 1 = ‘*not at all*’ to 5 = ‘*always*’. The items demonstrated face validity.

#### 2.2.3. Mental Health Stigma

The established Stigma and Self Stigma Scales (SASS; Docksey et al., 2022) were used to assess participants’ degree of mental health anticipated stigma and self-stigma. The SASS is a 42-item measure designed to assess multiple aspects of stigmatic beliefs about mental health problems for individuals with or without a history of mental health problems (Docksey et al., 2022; Gray et al., 2023). The SASS comprises six domains to assess stigma to others, social distance, anticipated stigma, self-stigma, avoidant coping, (lack of) help-seeking behaviors and a separate social desirability scale (Docksey et al., 2022). Based on the aims of the present study, the Anticipated Stigma subscale (e.g., *If I had a mental disorder, I would worry other people would think that I am weak*) and the Self-Stigma subscale (e.g., *If I had a mental disorder, I would feel ashamed*) were used. The use of these two subscales is also consistent with prior research (Aird et al., 2025; Batterham et al., 2024; Docksey et al., 2022). Each of the two subscales comprised 6-items. Participants rated each item on a 5-point Likert scale ranging from 0 = *strongly disagree* to 4 = *strongly agree*, with total possible subscale scores ranging from 0 to 24. Higher scores are indicative of increased anticipated stigma and self-stigma.

Research has demonstrated acceptable 4-week test–retest reliability for the Anticipated stigma (*r* = 0.72) and Self-Stigma subscales (*r* = 0.76; Docksey et al., 2022). Face validity was confirmed by a team of clinical and forensic psychologists reviewing the final items (Docksey et al., 2022). The Anticipated stigma and Self-Stigma subscales demonstrated strong internal reliability in the present study (Cronbach’s α = 0.84 and α = 0.88, respectively).

### 2.3. Procedure

The Edith Cowan University (ECU) Human Research Ethics Subcommittee approved the research (Reference: 2024-05264-DICKSON). Following approval, a participant information sheet and optional information video outlining the study purpose and participation requirements were provided at the beginning of the online survey. Participants indicated informed consent by ticking a box before proceeding. Those who declined consent were directed to the end of the survey. Participation was entirely voluntary.

ECU students aged 18 years and over were recruited via Engagement and Learning in Psychology through Active Research Participation (ELPARP) scheme. Students were awarded credit points (5%) towards their modules to acknowledge participation. Additionally, the study was advertised through social media platforms (Facebook/Instagram), online tech forums, AI and technology conference meetups, and ECU internal student news platforms for non-ELPARP participants and community members. All responses were anonymous. The online Qualtrics study was open from March 2024–August 2024. Measures were counterbalanced to control for presentation order effects and fatigue effects (Schuman & Presser, 1996).

### 2.4. Data Analyses

Analyses were conducted using IBM SPSS version 29 (IBM Corp., 2023). The mediation analyses were conducted in SPSS using PROCESS v4.2 (Hayes, 2022), using Model 4. Bootstrapped confidence intervals were computed using 5000 samples. Standardized coefficients are reported.

### 2.5. Data Screening

Prior to conducting the analyses to test our predictions, all data were initially screened following guidelines by Tabachnick and Fidell (2007). Data were reviewed in SPSS to ensure accuracy of entry, and no missing values were identified in the set. All variables were normally distributed and parametric assumptions were met.

The assumptions of normality and linearity were met with all study variables and all skewness and kurtosis values fell below the recommended thresholds for acceptable normality, with skewness and kurtosis within the range of ±2 (Schmider et al., 2010). Multiple regression assumption checks were satisfied showing homoscedasticity amongst study variables, with no multicollinearity and no multivariate outliers.

### 2.6. Power Calculations

A priori power calculations were conducted using G*Power software (version 3.1.9.6). Based on Cohen’s (1988) guidelines to ensure sufficient sample sizes for detecting at least medium effect sizes with a power of 0.80 and an alpha level of *p* < 0.05, a minimum of 55 participants were required for the main mediation analyses (*f*^2^ = 0.15). The final sample size (*n* = 73) was sufficient to detect medium effects in the mediation analyses, including three variables (the predictor, mediator and covariate). The sample size was also sufficient to detect medium to large effects for the correlation analyses (two-tailed).

## 3. Results

### The Relationships Between ChatGPT Use and Stigma

Means, standard deviations and Pearson’s *r* correlations between the main study variables for ChatGPT use, perceived effectiveness, anticipated stigma, and self-stigma are shown in Table 1 below.

As can be seen in Table 1, ChatGPT usage had a significant medium to large positive correlation with perceived effectiveness, but not with anticipated stigma nor with self-stigma. Perceived effectiveness showed a significant small to medium negative correlation with anticipated stigma but not with self-stigma. As would be expected, anticipated stigma and self-stigma showed a significant and strong positive correlation. Age (mean = 29.9 years, range 18–61, SD = 9.89) had a small significant negative correlation with anticipated stigma (*r* = −0.24) and self-stigma (*r* = −0.23) but did not significantly correlate with ChatGPT usage nor perceived effectiveness (*p*s > 0.05). Finally, gender had no significant correlations with any of the main study variables.

There was no significant difference between the university sample and the community sample on the main study variables (all *p*s > 0.05), indicating the suitability of combining the samples. Next, we tested the two hypothesized mediation predictions.

Does Perceived Effectiveness Mediate the Relationship Between ChatGPT Use and Anticipated stigma?

**Figure 1:**
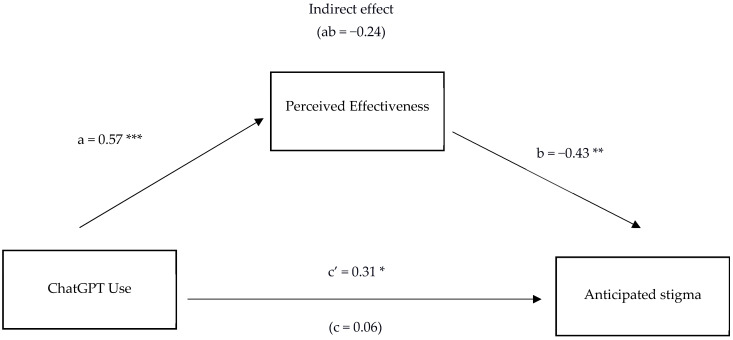
Mediation Model Showing the Relationship Between ChatGPT Use and Anticipated stigma via Perceived Effectiveness. Note. *N* = 73. Numerical values are standardized coefficients. * *p* < 0.05. ** *p* < 0.01. *** *p* < 0.001.

Results of the mediation analysis are shown below in Figure 1.

As seen, perceived effectiveness indirectly and significantly mediated the relationship between ChatGPT usage and anticipated stigma. The indirect effect was medium sized and significant, with ab = −0.24, BCa 95% CIs −0.41 to −0.09. Partial mediation occurred as the direct c’ path was also a medium effect size and significant (0.31). Age was included in the model as a covariate as prior correlational analyses found it to be significantly correlated with anticipated stigma. In our model, age was not associated with perceived effectiveness (0.02, *p* = 0.833) but was significantly associated with anticipated stigma (−0.23, *p* = 0.036).

Does Perceived Effectiveness Mediate the Relationship Between ChatGPT Use and Self-Stigma?

**Figure 2:**
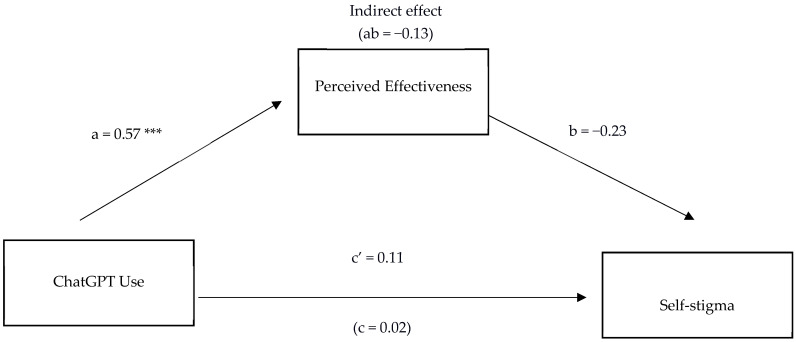
Mediation Model Showing the Relationship Between ChatGPT Use and Self Stigma via Perceived Effectiveness. Note. *N* = 73. Numerical values are standardized coefficients. *** *p* < 0.001.

Next, we investigated whether Perceived Effectiveness mediates the relationship between ChatGPT usage and self-stigma. As can be seen in Figure 2 below, perceived effectiveness did not significantly mediate the relationship between ChatGPT usage and self-stigma. As such, there was no significant indirect effect (ab = −0.13, BCa 95% CIs −0.29 to 0.03). As in the previous model, the direct path between ChatGPT and perceived effectiveness showed a significant large effect size (0.57, *p* < 0.001) but the b path between perceived effectiveness and self-stigma, although approaching significance, was non-significant (−0.23, *p* = 0.103). Both direct c and c’ paths between ChatGPT and self-stigma were non-significant. As age was significantly correlated with self-stigma, it was included as a covariate within the model. Age was not significantly associated with perceived effectiveness (0.02, *p* = 0.833) and although not significant with self-stigma was approaching significance (−0.23, *p* = 0.053).

## 4. Discussion

Overall, we aimed to study the relationships between ChatGPT usage for mental health, perceived effectiveness, and anticipated stigma and self-stigma. We first examined whether ChatGPT use for personal mental health support was significantly associated with anticipated stigma and self-stigma in relation to mental health difficulties. Next, we investigated whether the perceived effectiveness of ChatGPT in helping people with their own mental health explained, at least in part, the relationship between ChatGPT use and stigma. Contrary to our predictions, ChatGPT use was not significantly associated with either form of stigma, but ChatGPT usage and its perceived effectiveness for mental health support were significantly positively correlated. Contrasting results were found in the mediation analyses. As predicted, the perceived effectiveness of ChatGPT significantly mediated the relationship between ChatGPT usage and anticipated stigma. The mediation here had a suppressing effect (see MacKinnon et al., 2000), i.e., greater usage of ChatGPT and greater perceive effectiveness of it for mental health difficulties was associated with reduced anticipated stigma. That is to say, our findings suggest a relationship between those participants who perceive it as useful and lower reported anticipated stigma. Why might this be so? Speculatively, perceptions of usefulness may incline these participants to both heed and act on advice given by ChatGPT, and it may be that these participants are specifically looking for solutions to reduce their stigma. *Contrary* to expectations, perceived effectiveness did not significantly mediate the relationship between ChatGPT use and self-stigma, though the effect approached significance (i.e., CI not crossing zero). Implications of these findings are discussed below.

The significant positive relationship found between ChatGPT use and perceived effectiveness indicates that for participants who have used or are currently using AI, it is perceived as a useful tool for helping with their mental health issues. However, it is not known if participants believed AI would be useful prior to usage. Contrary to prediction, the lack of a significant correlation between ChatGPT use and anticipated stigma or self-stigma may suggest that ChatGPT was used to explore mental health issues, of which stigma is a component, but not the main driver of the participants’ usage. ChatGPT use and perceived effectiveness were not significantly associated with age. These non-significant findings suggest that using AI chatbots for mental health support may not be limited to a younger generation of university students but may instead reflect a broader trend across undergraduate student age groups.

The significant mediation findings highlight the importance of perceptions and beliefs about ChatGPT’s effectiveness in explaining the relationship between ChatGPT usage and anticipated stigma. Specifically, increased perceived effectiveness in ChatGPT use for mental health concerns was associated with a reduction in anticipated stigma. The observation that the c′ path is greater than the c path when the mediated path is accounted for indicates that *perceived effectiveness* in ChatGPT usage has a protective or buffering effect against the association between the *usage* of ChatGPT and anticipated stigma.

Given that a significant mediational effect was found for anticipated stigma, it was curious why no indirect mediation effect was found for self-stigma, although it was approaching significance. Speculatively, it is possible that a larger sample may detect a significant mediation effect in relation to self-stigma. Conceptually, and speculatively, self-stigma may reflect a more stable and internalized self-concept, thus being less amenable to change than anticipated stigma. In contrast, anticipated stigma may fluctuate more readily due to a greater degree of uncertainty regarding expectations, relative to self-stigma, thus being potentially more amenable to change in relation to digital interactions with AI chatbots. Past research indicates that self-stigma reduction interventions typically involve weekly structured psychoeducational Cognitive Behavioral Therapy (CBT) sessions (Fung et al., 2011; Lucksted et al., 2011) to address the multi-faceted complexity of tackling the schemas and core beliefs that constitute internalized self-stigma (Mills et al., 2020). Future research may benefit from examining whether addressing anticipated stigma, which appears more amenable to change, provides an entry point for addressing self-stigma.

The findings highlight that it is not merely the use of ChatGPT per se, but its perceived effectiveness, which is critical in understanding the effectiveness of ChatGPT in addressing anticipated mental health stigma. As individuals openly converse with ChatGPT about their mental health, they may receive empathetic and supportive responses, which in turn reinforce their perception of its effectiveness. The positive experiences and judgment-free environment that ChatGPT offers may help to normalize mental health discussions in an anonymous and safe space, thereby reducing perceptions of social prejudice. In other words, these interactions may help users feel less worried about being potentially judged if they were to disclose a mental health concern with others. Furthermore, some stigma pathways can be conceptualized as an internalization process, where initial concerns can manifest into more deeply seated core beliefs (Lannin et al., 2015). ChatGPT may be particularly effective at the early stages of this process, helping to alleviate anticipated stigma concerns before they internalize into self-stigma.

Overall, our results support past and recent findings, highlighting the increasing presence and positive perception of AI chatbots in mental health spaces (Haque & Rubya, 2023). Chatbots can serve as 24/7, judgment-free, low-cost companions, offering empathetic advice and enabling users to discuss their concerns comfortably (Ayers et al., 2023). Research demonstrates that university students more frequently cited cost, time, and stigma as barriers to accessing traditional mental health services than using chatbot-based support (Rackoff et al., 2025), which aligns with evidence that online technological interventions can reduce anticipated stigma (Rodríguez-Rivas et al., 2021). A recent related study found that clinicians perceived ChatGPT as a consistent, personalized, and therapeutically effective tool for supporting ADHD interventions (Berrezueta-Guzman et al., 2024). Taken together with the present findings, showing that users who perceive ChatGPT as effective also report reduced anticipated stigma, we suggest that future research develop and validate robust measures of this emerging construct of perceived effectiveness, both for ChatGPT specifically and for AI chatbots and mental health-related AI technologies more broadly.

Both Rackoff et al. (2025) and Gbollie et al. (2023) reported that 4.5% of their undergraduate participant sample had used AI chatbots for specific mental health support. In comparison, 73 participants (18.4%) in our study (from a larger sample of 397) reported using ChatGPT for their mental health purposes. The self-selected sampling may have resulted in a response bias, including a greater proportion of people with interests in AI-oriented uses for mental health. However, AI and ChatGPT is also garnering increasing media coverage and therefore familiarity with most people, so speculatively the greater proportion in our study may represent a longer-term upward trend in university students using AI-assisted mental health supports.

The quality, and therefore perceptions, of AI chatbots such as ChatGPT will likely continue to improve with the rapid development of the global AI industry. As a result, it is feasible to predict that undergraduate students’ mental health stigma may become increasingly amenable to both perceptions of chatbot effectiveness and increased usage, potentially encouraging greater uptake.

### Practical Implications

In most countries, access to specialist mental health services is either costly or, if free or relatively inexpensive, involves lengthy waitlists (Keynejad et al., 2021; Thomas et al., 2021). Therefore, digital options like ChatGPT may allow people to explore issues before seeking therapy or even be used as an adjunct to therapy, potentially easing therapist demand and providing quick, convenient support at unsociable hours (López et al., 2019). There are ethical issues with this approach though, as some users may rely on ChatGPT to provide support to help navigate complex mental health issues, that require professional and evidence-based interventions. Therefore, this research may help inform digital mental health literacy programs by encouraging users to remain critical when engaging with AI chatbot mental health content. Some AI platforms have shifted from a humanitarian open-source model toward profit-driven interests (Widder et al., 2023), raising questions about how data is collected, stored and used. The ethics of AI in sensitive domains such as accessing mental health support warrants further inquiry. Moreover, a regulatory gap is highlighted by the absence of standardized benchmarks for evaluating AI’s impact on mental health (Maslej et al., 2024). However, AI chat bots are free, accessible, and anonymous support may particularly appeal to undergraduate students, given their common barriers, such as limited financial resources, time constraints, and the stress associated with academic pressures. Still, AI chatbots may help these individuals better identify their needs, help reduce some initial stigma concerns and increase active help seeking with human professionals. This research may inform university student well-being policy and initiatives by raising awareness of AI chatbot mental health support and signposting students towards professional and evidence-based support for those who may already be engaging with ChatGPT, and other AI chatbots.

We recognize the challenges involved in monitoring and regulating AI chatbot technologies across international borders, particularly considering the World Health Organization’s recommendation that governments regulate AI at a local level (World Health Organization, 2025). Therefore, we recommend that AI companies provide standard mental health safety messaging that encourages users to treat advice with caution and seek professional support when needed and/or to work collaboratively with governments worldwide to provide clearer ethical guidelines and to ensure that mental health guidance is contextually appropriate and culturally specific. Moreover, it is important that mental health digital literacy is addressed within educational systems, with training on how to use AI tools in a responsible and informed manner.

Some key methodological considerations deserve comment. The sample was predominantly female, which may represent a sample bias. Given the aims of our research, the sample comprised predominantly undergraduate university students, which limits the generalizability of the findings more generally. The study’s cross-sectional design limits the ability to draw causal inferences. Further, it should be noted that because normative usage patterns for AI-based mental health support are largely unknown, we used broad categorical ranges to align with our research aims. While the mediation analyses provide insights into possible pathways, longitudinal research is needed to confirm these directional relationships. Moreover, new measures of perceived effectiveness of digital tools such as AI, and their usage need to be developed to reliably and validly capture these constructs and permit more sophisticated theoretical and statistical modeling.

There are several avenues for future research to pursue in this emerging area of mental health research. Although we surveyed ChatGPT use and participants’ perceptions of its effectiveness for their personal mental health issues, future research would benefit from determining the types of mental health issues participants are using ChatGPT for, and how they are using it. It would also be useful for future research to include measures of psychological symptoms, which may highlight where in the stigma internalization process, ChatGPT use is perceived as most effective. To gain deeper insight into why people use ChatGPT, qualitative studies and mixed methods studies may elucidate why university students and people from the wider community use ChatGPT for mental health support (e.g., what mental health issues are people using it for? What information or advice are people seeking? What are their specific stigma concerns?). Additionally, future research would benefit from a closer examination of whether there are differences between mental health difficulties, such as anxiety, depression, suicidal ideation in their ChatGPT usage or whether ChatGPT usage is generalizable across mental health conditions. Replication using larger student and community samples will help determine the stability and generalizability of our findings. Finally, future longitudinal study designs will help determine the direction of effects.

## 5. Conclusions

This research represents one of the first studies to investigate the associations between anticipated stigma, self-stigma, ChatGPT use and its perceived effectiveness. Our findings are promising in shedding some light on the relationships between these constructs. Still, further research, using psychometrically validated tools to assess the perceived effectiveness of ChatGPT awaits further investigation to advance this emerging field of research and to inform best practice in the use of AI for mental health purposes.

## Figures and Tables

**Table 1:** Descriptive Statistics and Pearson’s *r* Correlations Between Study Variables.

Variable	2.	3.	4.	M (SD)
1. ChatGPT usage	0.57 ***	0.07	−0.01	2.04 (1.01)
2. Perceived effectiveness	-	−0.25 *	−0.17	2.51 (0.96)
3. Anticipated stigma		-	0.80 ***	20.23 (6.74)
4. Self-stigma			-	19.51 (6.37)

Note. *N* = 73, * *p* < 0.05, *** *p* < 0.001.

## Data Availability

The original data presented in the study are available in Edith Cowan University Research Online Institutional Repository at https://doi.org/10.25958/cxnr-a008 (accessed on 5 October 2025).
